# Author Correction: Multi-phase-field simulation of microstructure evolution in metallic foams

**DOI:** 10.1038/s41598-021-95371-2

**Published:** 2021-08-05

**Authors:** Samad Vakili, Ingo Steinbach, Fathollah Varnik

**Affiliations:** 1 Interdisciplinary Centre for Advanced Materials Simulation (ICAMS), Ruhr-Universität Bochum, Universitätsstr. 150, 44801 Bochum, Germany; 2 Present Address: Max-Planck-Institut für Eisenforschung GmbH, Max-Planck-Straße 1, 40237 Düsseldorf, Germany

Correction to: *Scientific Reports* 10.1038/s41598-020-76766-z, published online 17 November 2020

The original version of this Article contained errors in Figure 8, where the bottom row image depicting the final state of time evolution of the foam structure was erroneously replaced by a duplication of the middle row.

The original Figure 8 and accompanying legend appear below.

**Figure 8:**
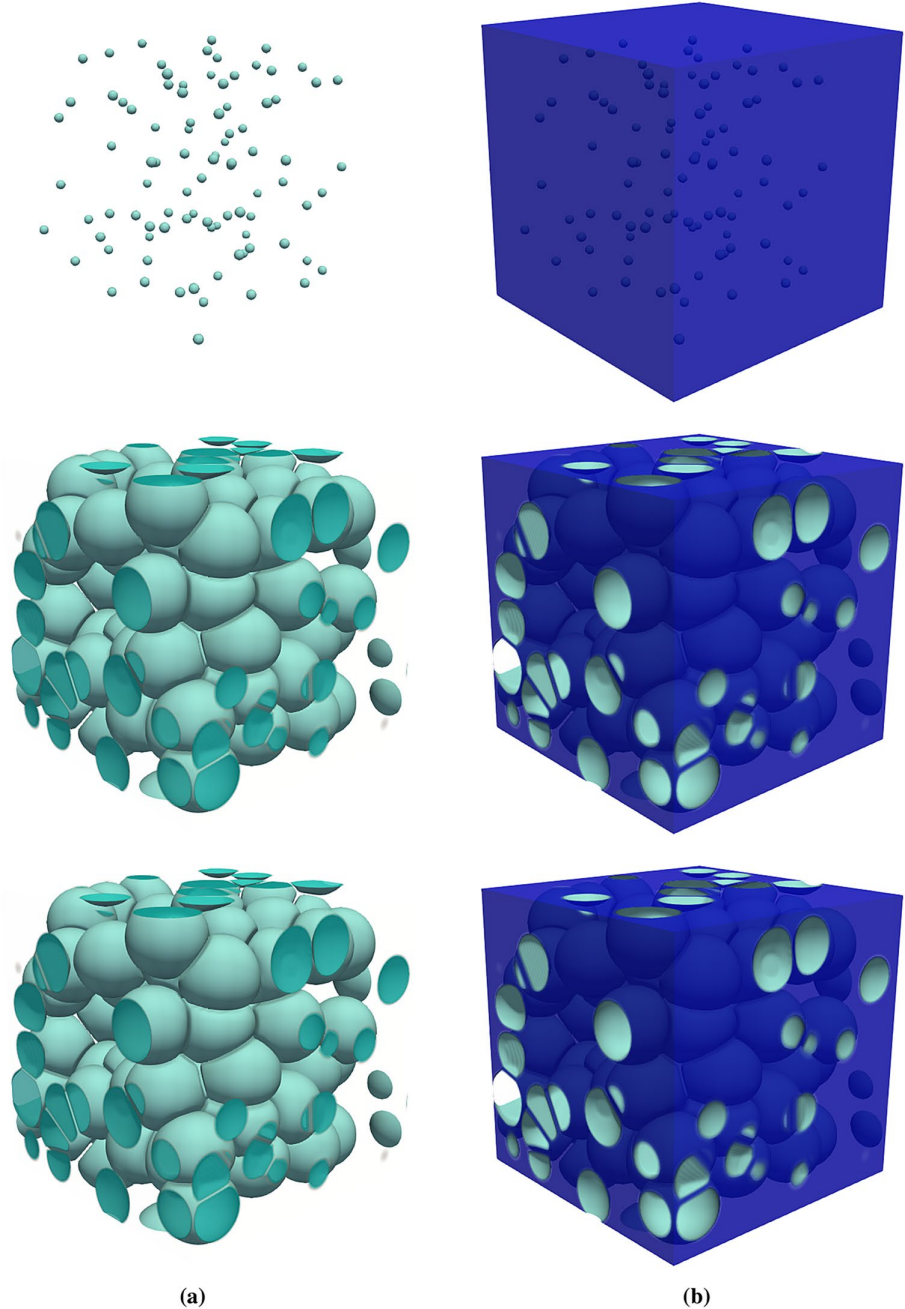
Result of 3D simulation for microstructure evolution of a foam representing bubbles in (**a**) and the liquid film around them in (**b**). Each raw, beginning from the top, corresponds to $t=0$, $1.5\times 10^4$, and $5\times 10^4\Delta t$. The system size is $300\times 300\times 300$, in the units of numerical resolution. The interface width is set to $\eta =6\,\Delta x$. The initial density of each bubble is assigned from the range of $\rho _\alpha (t=0) = 3.24-3.6$ and the density of the liquid is constant throughout the simulation, $\rho _{\mathrm{l}}$. Thus, the density ratio is around $\rho _{\mathrm{l}}/\rho _{\mathrm{g}} \approx 10,000$.

The original Article has been corrected.

