# Supplementary material for: Multi-phase-field simulation of microstructure evolution in metallic foams
Source: Sci Rep. 2020 Nov 17;10:19987. doi: 10.1038/s41598-020-76766-z (PMC7673141; doi:10.1038/s41598-020-76766-z)
Supplement: Supplementary file 1 — Supplementary Information. [file 41598_2020_76766_MOESM1_ESM.pdf]

Multi-phase-field simulation of microstructure evolution in metallic foams

Samad Vakili[1,a,b], Ingo Steinbach[2,a], and Fathollah Varnik[3,a]

[a]Ruhr-Universität Bochum, Interdisciplinary Center for Advanced Materials Simulation (ICAMS), Universitätsstr. 150, 44801 Bochum, Germany
[b]Current Address: Max-Planck-Institut für Eisenforschung GmbH, Max-Planck-Straße 1, 40237 Düsseldorf, Germany
[1]samad.vakili@rub.de
[2]ingo.steinbach@rub.de
[3]fathollah.varnik@rub.de (Corresponding author)

Appendix A Derivation of pressure tensor

In order to obtain a closed expression for the pressure tensor, instead of $\mathcal{F}$, we start from a slightly different free energy functional,

$$\tilde{\mathcal{F}} = \int_\Omega \Big(\mathcal{L}\big(\{\phi\},\{\nabla\phi\}\big) + \lambda(t)\big(\sum_{\alpha=1}^{N}\phi_\alpha - 1\big)\Big)dV, \tag{A.1}$$

where $\lambda(t)$ is a Lagrange multiplier. As seen from a comparison with Eq. (1), the integral over the first term is the usual free energy functional, $\mathcal{F}$. The second term in Eq. (1) ensures that the sum of all phase fields is conserved at any point in space.

To simplify notation in the following derivation, we introduce $\tilde{\mathcal{L}} \equiv \mathcal{L}\big(\{\phi\},\{\nabla\phi\}\big) + \lambda(t)\big(\sum_{\alpha=1}^{N}\phi_\alpha - 1\big)$. With this definition, Eq. (A.1) takes the form

$$\tilde{\mathcal{F}} = \int_\Omega \tilde{\mathcal{L}}\big(\{\phi\},\{\nabla\phi\}\big)dV. \tag{A.2}$$

At equilibrium,

$$\frac{\partial\tilde{\mathcal{L}}}{\partial\phi_\alpha} - \frac{d}{dx_i}\frac{\partial\tilde{\mathcal{L}}}{\partial\partial_i\phi_\alpha} = 0, \tag{A.3}$$

for $\alpha = 1, ..., N$. Similar to [19], the total derivative for $\tilde{\mathcal{L}}$ yields

$$\begin{aligned}\frac{d\tilde{\mathcal{L}}}{dx_j} &= \frac{\partial\tilde{\mathcal{L}}}{\partial x_j} + \sum_{\alpha=1}^{N}\Big(\frac{\partial\tilde{\mathcal{L}}}{\partial\partial_i\phi_\alpha}\partial_j\partial_i\phi_\alpha + \frac{\partial\tilde{\mathcal{L}}}{\partial\phi_\alpha}\partial_j\phi_\alpha\Big)\\ &= \frac{\partial\tilde{\mathcal{L}}}{\partial x_j} + \sum_{\alpha=1}^{N}\frac{d}{dx_j}\Big(\frac{\partial\tilde{\mathcal{L}}}{\partial\partial_i\phi_\alpha}\partial_j\phi_\alpha\Big),\end{aligned} \tag{A.4}$$

where $\partial\tilde{\mathcal{L}}/\partial\phi_\alpha$ is replaced by $(d/dx_i)(\partial\tilde{\mathcal{L}}/\partial\partial_i\phi_\alpha)$ and then the product rule is used to derive the second line. Equation (A.4) is reordered to

$$\frac{\partial\tilde{\mathcal{L}}}{\partial x_j} = \frac{d}{dx_i}\Bigg(\tilde{\mathcal{L}}\delta_{ij} - \sum_{\alpha=1}^{N-1}\frac{\partial\tilde{\mathcal{L}}}{\partial\partial_i\phi_\alpha}\partial_j\phi_\alpha\Bigg) \equiv \nabla\cdot\mathbf{P}. \tag{A.5}$$

Similar to pressure, the tensor $\mathbf{P}$ introduced above has the dimension of energy density. Moreover, it follows from Eq. (A.5) that this tensor is divergence free if the Lagrange function $\mathcal{L}$ obeys translational invariance, i.e., if it does not explicitly depend on position. The close connection between momentum conservation and translational invariance then implies the existence of a divergence free pressure tensor, which we identify as $\mathbf{P}$ [27]. To proceed further, we express $\tilde{\mathcal{L}}$ in terms of $\mathcal{L}$ and Lagrange multiplier to obtain ($\mathbf{I}$ is the identity tensor),

$$\nabla\cdot\mathbf{P} = \nabla\cdot\Big(\mathcal{L}\,\mathbf{I} - \sum_{\alpha=1}^{N}\frac{\partial\mathcal{L}}{\partial\nabla\phi_\alpha}\nabla\phi_\alpha\Big) + \lambda(t)\sum_{\alpha=1}^{N}\nabla\phi_\alpha. \tag{A.6}$$

The Lagrange multiplier, $\lambda(t)$, can be expressed via derivatives of $\mathcal{L}$ by using the Euler-Lagrange equations (A.3). The result is

$$\lambda(t) = -\frac{1}{N}\sum_{\beta=1}^{N}\Big(\frac{\partial\mathcal{L}}{\partial\phi_\beta} - \nabla\cdot\frac{\partial\mathcal{L}}{\partial\nabla\phi_\beta}\Big). \tag{A.7}$$

Inserting this relation in Eq. (A.6) yields

$$\begin{aligned}\nabla\cdot\mathbf{P} &= \frac{N}{N}\sum_{\alpha=1}^{N}\Big(\frac{\partial\mathcal{L}}{\partial\phi_\alpha}\nabla\phi_\alpha - (\nabla\cdot\frac{\partial\mathcal{L}}{\partial\nabla\phi_\alpha})\nabla\phi_\alpha\Big) - \frac{1}{N}\sum_{\alpha=1}^{N}\sum_{\beta=1}^{N}\Big(\frac{\partial\mathcal{L}}{\partial\phi_\beta} - \nabla\cdot\frac{\partial\mathcal{L}}{\partial\nabla\phi_\beta}\Big)\nabla\phi_\alpha\\ &= \frac{1}{N}\sum_{\alpha=1}^{N}\sum_{\beta=1}^{N}\Big\{\Big(\frac{\partial\mathcal{L}}{\partial\phi_\alpha} - \nabla\cdot\frac{\partial\mathcal{L}}{\partial\nabla\phi_\alpha}\Big) - \Big(\frac{\partial\mathcal{L}}{\partial\phi_\beta} - \nabla\cdot\frac{\partial\mathcal{L}}{\partial\nabla\phi_\beta}\Big)\Big\}\nabla\phi_\alpha\\ &= \frac{1}{N}\sum_{\alpha=1}^{N}\sum_{\beta=1}^{N}\Big\{\frac{\delta\mathcal{F}}{\delta\phi_\alpha} - \frac{\delta\mathcal{F}}{\delta\phi_\beta}\Big\}\nabla\phi_\alpha.\end{aligned} \tag{A.8}$$

In order to evaluate forces arising from divergence of the pressure tensor in terms of the model parameters, the free energy density, $\mathcal{L}$, must be specified. A standard choice is [19]

$$\mathcal{L}=\sum_{\alpha=1}^{N-1}\sum_{\beta=\alpha+1}^{N}\Big(-\frac{W_{\alpha\beta}^2}{2}\nabla\phi_\alpha\cdot\nabla\phi_\beta+\frac{\gamma_{\alpha\beta}}{2}|\phi_\alpha\phi_\beta|-\big[h(\phi_\alpha)p_\alpha(\rho_\alpha)+h(\phi_\beta)p_\beta(\rho_\beta)\big]\Big), \tag{A.9}$$

where $W_{\alpha\beta}^2$ is the analog of square-gradient coefficient for the case of multiple phases, $\gamma_{\alpha\beta}$ tunes the strength of potential energy and p_α and p_β are bulk pressures within the phases α and β, respectively. The sum in square brackets is an average pressure with h playing the role of an interpolation function. It is possible to express W_{ij}^2 and γ_{ij} in terms of interface energy and thickness, $\sigma_{\alpha\beta}$ and η, respectively. For this purpose, we consider force balance at a planar interface and obtain (see Appendix A.1),

$$\gamma_{\alpha\beta} = \frac{8\sigma_{\alpha\beta}}{\eta}, \tag{A.10}$$

$$W_{\alpha\beta}^2 = \frac{8\sigma_{\alpha\beta}\eta}{\pi^2}. \tag{A.11}$$

Inserting Eqs. (A.10) and (A.11) in Eq. (A.9), one arrives at

$$\mathcal{L}=\sum_{\alpha=1}^{N-1}\sum_{\beta=\alpha+1}^{N}\Big(-\frac{4\sigma_{\alpha\beta}\eta}{\pi^2}\nabla\phi_\alpha\cdot\nabla\phi_\beta+\frac{4\sigma_{\alpha\beta}}{\eta}|\phi_\alpha\phi_\beta|-h(\phi_\alpha)p_\alpha(\rho_\alpha)-h(\phi_\beta)p_\beta(\rho_\beta)\Big). \tag{A.12}$$

Guidance for a reasonable choice of the interpolation function h can be obtained by considering a spherical bubble in equilibrium with the surrounding liquid. One can then verify that the following choice of the function h satisfies the force balance condition for a spherical bubble (see Appendix A.2),

$$h(\phi_\alpha)=\frac{1}{\pi}\Big(2(2\phi_\alpha-1)\sqrt{\phi_\alpha(1-\phi_\alpha)}+\arcsin(2\phi_\alpha-1)+\frac{\pi}{2}\Big). \tag{A.13}$$

Using Eq. (A.12), divergence of the pressure tensor can be expressed in terms of model parameters,

$$\begin{aligned}\nabla\cdot\mathbf{P} &= \sum_{\alpha=1}^{N}\sum_{\beta=1}^{N}\frac{1}{N}\Big\{\Big(\sum_{\xi=1,\xi\neq\alpha}^{N}-\frac{4\sigma_{\alpha\xi}\eta}{\pi^2}\big(\frac{\pi^2}{\eta^2}\phi_\xi+\nabla^2\phi_\xi\big)+\sum_{\xi=1,\xi\neq\beta}^{N}\frac{4\sigma_{\beta\xi}\eta}{\pi^2}\big(\frac{\pi^2}{\eta^2}\phi_\xi+\nabla^2\phi_\xi\big)\Big)+\\ &\quad (N-1)\Big(p_\alpha\frac{\partial h}{\partial\phi_\alpha}-p_\beta\frac{\partial h}{\partial\phi_\beta}\Big)\Big\}\nabla\phi_\alpha\\ &= \frac{4\eta}{\pi^2 N}\sum_{\alpha=1}^{N}\sum_{\beta=1}^{N}\Big\{\sum_{\xi=1}^{N}(\sigma_{\beta\xi}-\sigma_{\alpha\xi})I_\xi+\frac{(N-1)\pi^2}{4\eta}\Big(p_\alpha\frac{\partial h}{\partial\phi_\alpha}-p_\beta\frac{\partial h}{\partial\phi_\beta}\Big)\Big\}\nabla\phi_\alpha.\end{aligned} \tag{A.14}$$

In Eq. (A.14), $I_\xi=\nabla^2\phi_\xi+(\pi\phi_\xi/\eta)^2$ reflects curvature effects [33]. The second bracket on the rhs of Eq. (A.14) is the contribution of hydrostatic pressure to the driving force.

Equation (A.14) becomes relatively simple for the special case of a two-phase system. With $\phi_\alpha=\phi$ and $\phi_\beta=1-\phi$ one then obtains,

$$\nabla\cdot\mathbf{P}=\Big((p_\alpha-p_\beta)\frac{\partial h}{\partial\phi}-\gamma_{\alpha\beta}(\frac{1}{2}-\phi)+W_{\alpha\beta}^2\nabla^2\phi\Big)\nabla\phi. \quad \text{(two-phase system)} \tag{A.15}$$

Consider that Eq. (A.15) is obtained in terms of $W_{\alpha\beta}^2$ and $\gamma_{\alpha\beta}$ and without applying Eqs. (A.10) and (A.11). Note also that the interpolation function h appears only in a product with pressure difference between the two phases. As a consequence, h plays no role in force balance if hydrostatic pressure does not vary across the interface. An example is the equilibrium condition for two phases separated by a planar interface, which we discuss below.

A.1 Planar interface

For a planar interface, it directly follows from the force balance condition at equilibrium ($\nabla\cdot\mathbf{P}=\mathbf{0}$) that the component of pressure tensor along the direction normal to the interface is spatially constant. This implies $p_\alpha=p_\beta$ [19]. Inserting this information into the rhs of Eq. (A.15), the term containing the interpolation function drops and one obtains,

$$-\gamma_{\alpha\beta}\Big(\frac{1}{2}-\phi\Big)+W_{\alpha\beta}^2\frac{\partial^2\phi}{\partial x^2}=0, \tag{A.16}$$

where we assumed that the interface is normal to the x-direction. For the double obstacle potential used in the present study, the interface profile is given by [25],

$$\phi(x) = \begin{cases} 1 & x \leq -\dfrac{\eta}{2} \\ \dfrac{1}{2} - \dfrac{1}{2}\sin\Big(\dfrac{\pi}{\eta}x\Big) & -\dfrac{\eta}{2} \leq x \leq \dfrac{\eta}{2} \\ 0 & x \geq \dfrac{\eta}{2}, \end{cases} \tag{A.17}$$

where η is the interface width. Substitution of Eq. (A.17) into Eq. (A.16) gives

$$\gamma_{\alpha\beta} = \frac{\pi^2 W_{\alpha\beta}^2}{\eta^2}. \tag{A.18}$$

On the other hand, the specific interface free energy is given by integral over the free energy density, $\mathcal{L}$, Eq. (A.9), excluding the pressure term which contains the bulk free energy. It reads

$$\sigma_{\alpha\beta} = \int_{-\infty}^{\infty} \Big(\frac{W_{\alpha\beta}^2}{2}\Big(\frac{\partial\phi}{\partial x}\Big)^2 + \frac{\gamma_{\alpha\beta}}{2}\phi(1-\phi)\Big)dx = \int_{-\infty}^{\infty} W_{\alpha\beta}^2\Big(\frac{\partial\phi}{\partial x}\Big)^2 = \frac{\pi^2 W_{\alpha\beta}^2}{8\eta}, \tag{A.19}$$

where we used Eqs. (A.16) and (A.17) in the last steps. Combining Eqs. (A.18) and (A.19), the model parameters can be expressed in terms of surface free energy and interface thickness, as given above in Eqs. (A.10) and (A.11).

A.2 A single sphere

For a single sphere of phase α in equilibrium with the surrounding medium, β, the radial symmetry of the problem can be used to write $\phi \equiv \phi(r)$, with r being the distance from center of the sphere. As a consequence, one also can write $\nabla^2\phi = \partial^2\phi/\partial r^2 + ((d-1)/r)\partial\phi/\partial r$, where d is dimensions of space. Substitution of this into Eq. (A.15) and applying the equilibrium condition yields,

$$(p_\alpha - p_\beta)\frac{\partial h}{\partial\phi} - \gamma_{\alpha\beta}\Big(\frac{1}{2} - \phi\Big) + W_{\alpha\beta}^2\Big(\frac{\partial^2\phi}{\partial r^2} + \frac{d-1}{r}\frac{\partial\phi}{\partial r}\Big) = 0. \tag{A.20}$$

Using Eq. (A.20), it is possible to obtain guidance regarding the choice of a reasonable interpolation function. For this purpose, we approximate $\phi(r)$ by the planar interface profile, Eq. (A.17), (replacing, of course, x by $r - R$, with the sphere radius R). Within this planar approximation, we find $\partial\phi/\partial r = -\pi/\eta\sqrt{\phi(1-\phi)}$ and $\partial^2\phi/\partial r^2 = (\pi/\eta)^2(1-2\phi)$. Inserting these expressions into Eq. (A.20) gives,

$$(p_\alpha - p_\beta)\frac{\partial h}{\partial\phi} = \frac{8\sigma_{\alpha\beta}(d-1)}{\pi r}\sqrt{\phi(1-\phi)}. \tag{A.21}$$

Recalling that the product $\phi(1-\phi)$ is non zero only for values of r in the interface region, one can replace r by the radius of sphere R. Further, using the Young-Laplace equation $p_\alpha - p_\beta = \sigma_{\alpha\beta}(d-1)/R$, Eq. (A.21) simplifies to

$$\frac{\partial h}{\partial\phi} = \frac{8}{\pi}\sqrt{\phi(1-\phi)}. \tag{A.22}$$

One can check that Eq. (A.22) is satisfied by

$$h(\phi) = \frac{1}{\pi}\Big(2(2\phi-1)\sqrt{\phi(1-\phi)} + \arcsin(2\phi-1) + \frac{\pi}{2}\Big). \tag{A.23}$$
